# A Study on the Safety and Efficacy of an Innovative Hydrophilic Dialysis Membrane

**DOI:** 10.3390/membranes15010030

**Published:** 2025-01-14

**Authors:** Francisco Maduell, Victor Joaquín Escudero-Saiz, Elena Cuadrado-Payán, Maria Rodriguez-Garcia, Miquel Gómez, Lida María Rodas, Néstor Fontseré, Maria del Carmen Salgado, Gregori Casals, Nayra Rico, José Jesús Broseta

**Affiliations:** 1 Nephrology and Renal Transplantation, Hospital Clínic de Barcelona, 08036 Barcelona, Spain; vjescudero@clinic.cat (V.J.E.-S.); ecuadrado@clinic.cat (E.C.-P.); mgomezu@clinic.cat (M.G.); lmrodas@clinic.cat (L.M.R.); fontsere@clinic.cat (N.F.); jjbroseta@clinic.cat (J.J.B.); 2 Biochemistry and Molecular Genetics Department-CDB, Hospital Clínic de Barcelona, 08036 Barcelona, Spain; mrodriguezg@clinic.cat (M.R.-G.); salgado@clinic.cat (M.d.C.S.); casals@clinic.cat (G.C.); nrico@clinic.cat (N.R.)

**Keywords:** dialysate albumin loss, dialyzer performance, hemodiafiltration, hydrophilic membrane

## Abstract

The dialysis membrane based on a hydrophilic polymer (Hydrolink NV) was designed to enhance the movement of adsorbed water at the blood–membrane interface, aiming to achieve antithrombogenic and antifouling effects. This study aimed to assess the performance and albumin loss of the Hydrolink NV dialyzer in hemodialysis (HD) and post-dilution hemodiafiltration (HDF) with different infusion flows (Qis) and compare it with the hydrophilic FX CorAL dialyzer in post-dilution HDF. A prospective study was carried out in 20 patients. Patients underwent five dialysis sessions with the same routine dialysis parameters: four sessions with the Toraylight NV 2.1 (HD, post-dilution HDF with 50, 75 or auto-substitution Qi) and one with the FX CorAL 800 (post-dilution HDF with auto-substitution Qi). The reduction ratios’ (RRs’) wide range of molecular weight molecules were assessed and the dialysate albumin loss was quantified. The lowest β_2_-microglobulin, indoxyl-sulfate, and p-cresyl sulfate RR values were observed with the Toraylight NV 2.1 in HD, and they improved progressively with an increased Qi, without differences being observed between the two dialyzers in auto-substitution. A different removal profile was observed in terms of myoglobin, kFLC, prolactin, α_1_-microglobulin, α_1_-acid glycoprotein, and λFLC, whose RRs also improved progressively with an increased Qi but were significantly higher with the Toraylight NV than the CorAL in the same convective condition. There were significant differences in the albumin dialysate losses, with the highest value obtained with the Toraylight NV in auto-substitution HDF, with more than 50% of patients surpassing 5 g per session. The Toraylight NV dialyzer has great potential for efficacy but should be used at the optimal convective volume (Qi not exceeding 75 mL/min or FF not exceeding 25%) to avoid excessive albumin loss.

## 1. Introduction

Currently, the most commonly used synthetic high-flux dialyzers are made of polysulfone and polyethersulfone. These materials are predominantly utilized in hemodialysis (HD) and online hemodiafiltration (HDF). Polyvinylpyrrolidone (PVP) has an impact on the priming procedure and saline circulation, which reduces protein fouling and platelet adsorption [1,2,3,4]. As a result, it can be stated that it lowers the risk of intradialytic coagulation, although it may increase the likelihood of adverse reactions [5]. A small percentage of patients may exhibit intolerance to synthetic dialyzers [4]. Consequently, cellulose dialyzers such as cellulose triacetate (CTA) [6,7] or polymethylmethacrylate (PMMA) membranes [8] have been reintroduced as an alternative to avoid these clinical complications.

The new-generation dialysis membrane, developed by Toray Medical in Tokyo, Japan, features a specific hydrophilic polymer called Hydrolink^®^ NV. This polymer is applied to the inner surface of the polysulfone material, achieving complete inhibition of platelet adhesion with a 100% surface coverage rate. This means that the surface is completely covered by a single layer of the new hydrophilic polymer and that platelet adhesion is nearly completely inhibited. The goal is to eliminate platelet adhesion even in the absence of heparin [9]. Preliminary Japanese studies have indicated the potential benefits of the Hydrolink NV membrane in terms of the antithrombotic activity, low stimulation of platelets and leukocytes, and improved removal of β2-microglobulin, without compromising the removal performance of regular solutes during dialysis [9,10,11].

Following similar hydrophilic principles, the new-generation helixone FX CorAL^®^ dialyzer series has been developed to improve hemocompatibility. This dialyzer features a membrane made from a blend of polysulfone and PVP, enhanced with small amounts of tocopherol to stabilize the blood-side surface [12]. This modification promotes the formation of a more robust hydrophilic layer and enhances the hemocompatibility of polysulfone membranes by increasing their antifouling abilities. As a result, there is less protein adsorption and lower coagulation activation [1,13,14]. In a previous study [15], our group observed that this hydrophilic helixone maintained the effectiveness and albumin loss achieved with the previous helixone generation, with the potential advantage of preventing adverse reactions.

The objective of this study was to prospectively compare both hydrophilic membranes by assessing the performance and albumin loss of the Hydrolink NV dialyzer in HD and post-dilution HDF at different infusion flows (Qis) and to compare it with the hydrophilic FX CorAL dialyzer in post-dilution HDF. The removal of a wide range of molecular weight molecules was assessed and the risk of hypoalbuminemia was evaluated through quantification of the dialysate albumin loss.

## 2. Materials and Methods

This prospective, single-center study involved 20 patients, comprising 14 men and 6 women, with a mean age of 71.2 ± 12 years (range: 53 to 83). All the participants were undergoing regular hemodialysis for an average of 75 ± 96 months (range: 3–311). The vascular accesses were autologous arteriovenous fistula (AVF) in 12 patients, tunneled catheter in 7, and prosthetic arteriovenous fistula in the other patient. The anticoagulation used was low-molecular-weight heparin in 55% of the patients and heparin sodium in 35%; the remaining 10% were dialyzed without heparin. The underlying renal diseases were nephroangiosclerosis (seven patients), chronic glomerulonephritis (four patients), diabetic nephropathy (three patients), interstitial nephritis (two patients), urologic etiology (two patients), systemic disease (one patient), and undiagnosed nephropathy (one patient). All the patients provided informed consent. This study was approved by the local ethics committee and was conducted according to the principles of the Declaration of Helsinki. The dialyzer characteristics are summarized in Table 1.

Each patient underwent five dialysis sessions with routine dialysis parameters in which only the dialyzer and infusion flow were modified, that is, four sessions with the Toraylight NV 2.1 and one with the FX CorAL 800 dialyzer, as follows:

Toraylight NV 2.1 in HD.
Toraylight NV 2.1 in post-dilution HDF with Qi 50 mL/min.
Toraylight NV 2.1 in post-dilution HDF with Qi 75 mL/min.
Toraylight NV 2.1 in post-dilution HDF with Qi in auto-substitution.
FX CorAL 800 in post-dilution HDF with Qi in auto-substitution.

The prescribed dialysis parameters were a dialysis buffer with bicarbonate, dialysate flow of 400 mL/min, blood flow (Qb) of 448 ± 11 mL/min (range: 400–450) and dialysis time of 289 ± 18 min (range: 240–300). The net fluid removal was set individually, depending on the patient’s clinical needs. All the patients were anuric, with a urine volume of <50 mL/day. Fresenius 5008 Cordiax or 6008 CAREsystem (Fresenius, Bad Homburg, Germany) dialysis monitors were used. The variables collected in each session were as follows: real duration, dialyzer, Qb, recirculation index measured by the temperature module, arterial and venous pressure, transmembrane pressure (TMP), initial and final hematocrit automatically measured by the BVM^®^ biosensor, initial and final body weights, volume of blood processed, and replacement volume.

Blood and dialysis fluid samples for the analyses were taken from each patient in the same dialysis session of the week. The laboratory measurements included the concentrations of urea (molecular weight [MW] 60 Da), creatinine (113 Da), ß_2_-microglobulin (11,800 Da), myoglobin (17,200 Da), kappa-free immunoglobulin light chains (κFLC, 22,500 Da), prolactin (23,000 Da), α_1_-microglobulin (33,000 Da), α_1_-acid glycoprotein (41,000 Da), lambda-free immunoglobulin light chains (λFLC, 45,000), and albumin (66,000 Da) in the serum at the beginning and at the end of each session to calculate the reduction ratio (RR) of these solutes. The protein-bound uremic toxins (PBUTs), p-cresyl sulfate (188 Da) and indoxyl sulfate (213 Da) were also evaluated. The final concentrations of ß_2_-microglobulin, myoglobin, κFLC, prolactin, α_1_-microglobulin, α_1_-acid glycoprotein, λFLC, p-cresyl sulfate (pCS), indoxyl sulfate (IS), and albumin were corrected for the degree of hemoconcentration and the volume of distribution (approximate extracellular volume) according to Bergström and Wehle [16]. The mean total convective volume (replacement volume plus weight loss rate), infusion flow (Qi), filtration ratio (FF), and percentage of convective volume related to the total blood processed were calculated. Throughout the treatment, a proportional part of the dialysis fluid was collected to quantify the albumin loss

Urea and creatinine were measured by molecular absorption spectrometry, albumin and β2-microglobulin were measured by immunoturbidimetry, and myoglobin and prolactin were measured by indirect enzyme immunoassay (EIA), and all of them were performed in an Atellica Solution analyzer (Siemens Healthineers, Tarrytown, NY, USA). α_1_-microglobuline, α_1_-acid glycoprotein, κFLC, and λFLC were measured by immunonephelometry using the BNII analyzer (Siemens Healthineers). Finally, IS and pCS were measured in the serum using liquid chromatography–mass spectrometry (LC-MS): a stock solution of IS and pCS was prepared at a concentration of 100 mg/mL in water and stored at −20 °C; working solutions were prepared by mixing and diluting the stock solutions in water to a final concentration of 100,000 ng/mL for each metabolite; seven-point calibration curves (100, 500, 1000, 2500, 5000, 10,000, 40,000 ng/mL) were prepared by diluting the working solution in water. For the quality control, two levels were prepared (750 and 3500 ng/mL); the IS stock solutions of indoxyl sulfate ^13^C_6_ and p-cresyl sulfate-d_7_ were prepared at a concentration of 20 µg/mL and a working solution was prepared at a final concentration of 2 µg/mL for each metabolite; for the quantitative measurement of IS and pCS, protein precipitation was performed on 50 µL of serum sample using 340 µL of methanol. We used IS-^13^C_6_ and pCS-d_7_ as internal standards, based on the commercial availability of isotopically labelled uremic toxins. The mobile phases were methanol (0.1% formic acid) and water (0.1% formic acid).

We used the global removal score (GRS) to evaluate the effectiveness of a removal dialyzer, including the RR of molecules with an MW ranging from 60 to 41,000 and considering the albumin RR as negative values, calculated with the following formula [17]:

$$\frac{{\mathrm{Urea}}_{\mathrm{RR}}+\beta_{2}-{\mathrm{microglobulin}}_{\mathrm{RR}}+{\mathrm{myoglobin}}_{\mathrm{RR}}+{\mathrm{prolactin}}_{\mathrm{RR}}+\alpha_{1}-{\mathrm{microglobulin}}_{\mathrm{RR}}+\alpha_{1}-{\mathrm{acid}\;\mathrm{glycoprotein}}_{\mathrm{RR}}-{\mathrm{albumin}}_{\mathrm{RR}}}{6}$$

The results are expressed as the arithmetic mean ± standard deviation. For the analysis of the statistical significance of quantitative parameters, Student’s *t*-test was used for paired data and ANOVA for repeated data, followed by Bonferroni’s post hoc comparison tests for the parametric data. The linear regression coefficient was used to model the relationship of the dialysate albumin loss (dependent variable) with respect to the convective volume or Qi (independent variables). A *p* < 0.05 was considered statistically significant. The analyses were performed using SPSS software version 23 (SPSS, Chicago, IL, USA) and the graphics were produced using Prism version 10 (GraphPad Software, Boston, MA, USA).

## 3. Results and Discussion

Among the 20 included patients, one moved to another city during the follow-up, so 19 patients completed the five sessions of the protocol, and all the dialysis sessions were performed without filter adverse reactions or notable clinical incidents.

There were no differences in the Qb, total blood processed, vascular access recirculation, initial weight, final weight, weight gain, initial and final hematocrit, arterial pressure, and venous pressure. As expected, differences in the TMP and convective volume were found in relation to the infusion flow used. Finally, a few differences were observed in the real session duration, that is, two or three minutes more in HD compared with the HDF treatments (Table 2).

### 3.1. Small-Sized Molecules

**Figure 1:**
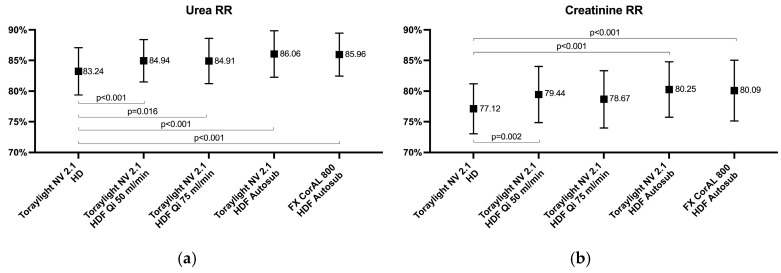
Comparison of the urea (**a**), MW 60, and creatinine (**b**), MW 113, reduction ratios in all the study situations.

The dialysis diffusive doses, as measured by the urea and creatinine RRs, were slightly lower in the HD modality than in all four HDF treatments (Figure 1).

### 3.2. Medium-Sized Molecules

**Figure 2:**
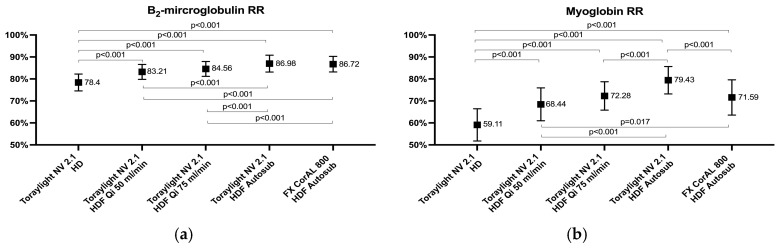

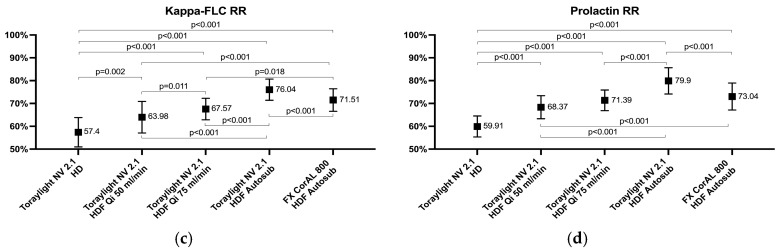
Comparison of the β_2_-microglobulin (**a**), MW 11,800, myoglobin (**b**), MW 17,200, kappa free immunoglobulin light chains (**c**), MW 22,500, and prolactin (**d**), MW 23,000, RRs in all the study situations.

The average values of the β2-microglobulin RRs ranged between 78% and 87%. The lowest value was observed for the Toraylight NV in HD and the β2-microglobulin RRs increased progressively with an increased Qi. There were no differences between the Toraylight NV and the FX CorAL in auto-substitution (Figure 2). A different removal profile was observed in solutes with a higher molecular weight. The myoglobin RRs increased progressively with an increased Qi, and the Toraylight NV, in the situation of maximum convective volume, was significantly higher than the FX CorAL (Figure 2). A similar profile was observed in the free kFLC and prolactin RRs. It also increased progressively with an increased Qi, and the Toraylight NV, in HDF auto-substitution, was again significantly higher than the FX CorAL (Figure 2).

**Figure 3:**
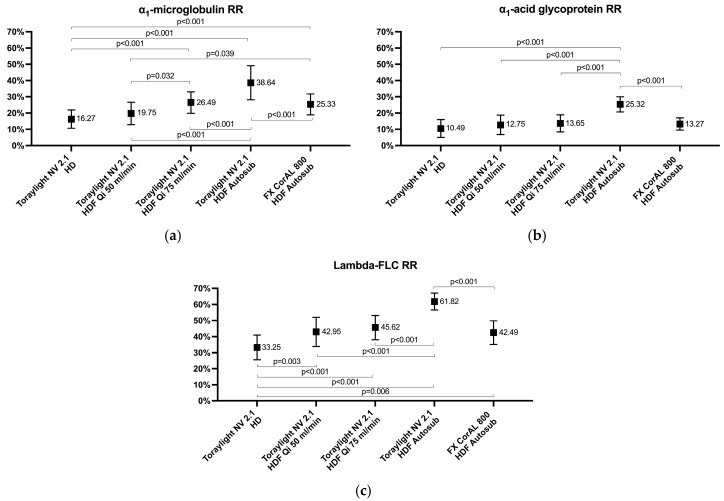
Comparison of the α1-microglobulin (**a**), MW 33,000, α1-acid glycoprotein (**b**), MW 41,000, and lambda free immunoglobulin light chains (**c**), MW 45,000, RRs in all the study situations.

In the high-molecular-weight range, the α1-microglobulin and α1-acid glycoprotein RRs values were between 10% and 40%. The α1-microglobulin and α1-acid glycoprotein RRs increased progressively with an increased Qi, and the Toraylight NV, in auto-substitution HDF, was again significantly higher than the FX CorAL (Figure 3). Finally, the free λFLC RRs also increased progressively with an increased Qi, and the Toraylight, in HDF auto-substitution, was again significantly higher compared to the FX CorAL (Figure 3).

### 3.3. Protein-Bound Uremic Toxins

**Figure 4:**
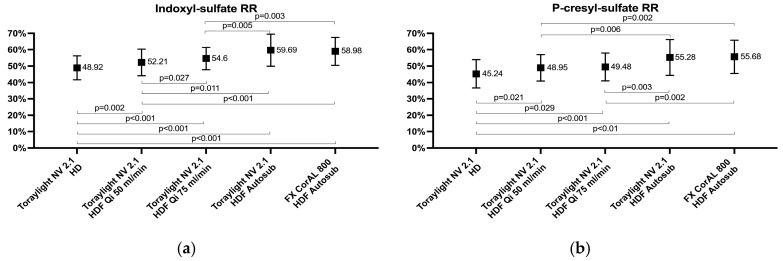
Comparison of the indoxyl sulfate (**a**) and p-cresyl sulfate (**b**) RRs in all the study situations.

The average values of the indoxyl sulfate and p-cresyl sulfate RRs ranged between 40% and 60%. The lowest value was observed for the Toraylight NV in HD and both PBUT RRs increased progressively with an increased Qi. There were no differences between the Toraylight 2.1 NV and the FX CorAL 800 in auto-substitution (Figure 4).

The RR of the total PBUT observed in this study was primarily due to the free IS and pCS removal. Although there was also a loss of albumin, approximately 10%, it may have contributed only slightly to the overall results as it involved the loss of PBUT in its complex form.

### 3.4. Albumin Loss in Blood and Dialysate

**Figure 5:**
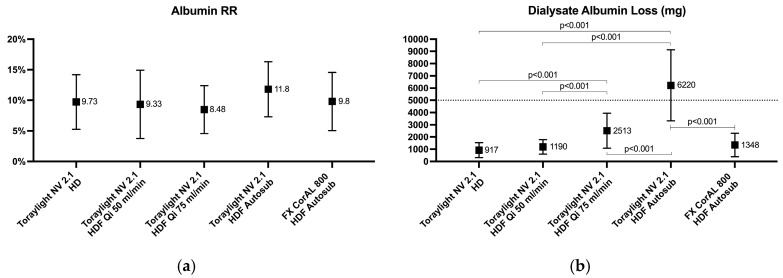
Comparison of the blood albumin reduction ratios (**a**) and dialysate albumin loss (**b**) in all the study situations.

No significant differences in the albumin RRs existed in any of the five study sessions. However, there were significant differences in the albumin dialysate losses. The highest losses were observed for the Toraylight NV in auto-substitution HDF, with a mean of 6220 ± 2899 mg. Interestingly, although there was a wide variation, in these sessions, more than 50% of patients had a dialysate albumin loss greater than 5 g. Nonetheless, the other four study situations had a mean albumin dialysate loss below 3 g (Figure 5).

**Figure 6:**
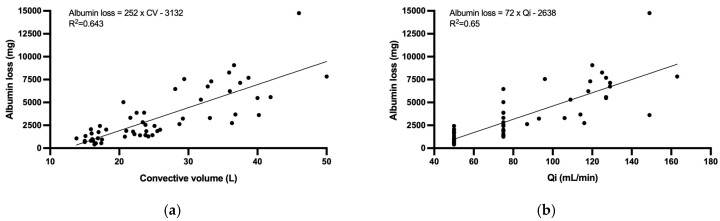
Correlation between the dialysate albumin loss and the convective volume (**a**) and infusion flow (**b**) under different HDF treatments with the Toraylight NV 2.1 dialyzer.

The dialysate albumin loss was positively correlated with the total convective volume (R^2^ = 0.643, *p* < 0.001) and with the Qi (R^2^ = 0.650, *p* < 0.001). Figure 6 illustrates these correlations with an equation for the trend line.

### 3.5. Global Removal Score

**Figure 7:**
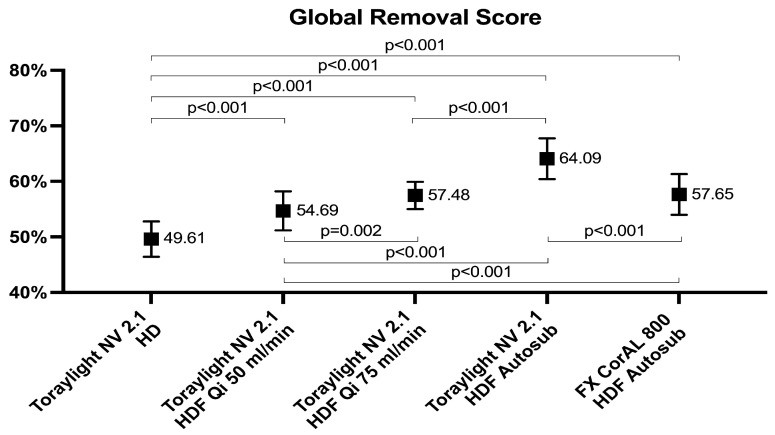
Comparison of the global removal score in all the study situations.

Significant differences were found between all the treatments evaluated. The GRS obtained with the Toraylight NV dialyzer in auto-substitution HDF was significantly higher than in the other study situations. The one obtained with the FX CorAL dialyzer in auto-substitution HDF was significantly higher than both the Toraylight NV in HD and HDF with Qi 50 mL/min. Finally, the GRS with the Toraylight NV in Qi 50 and Qi 75 mL/min were significantly higher than the Toraylight NV in HD treatment (Figure 7).

The present study’s results comparing these two new-generation hydrophilic membranes, the Toraylight NV versus the FX CorAL dialyzer, show that the Toraylight dialyzer’s removal efficiency under maximum convection conditions is slightly higher than its comparator, the FX CorAL. However, this difference is obtained with a dialysate albumin loss greater than 5 g, which could impact its safety in the medium or long term. On the other hand, when we compare the Toraylight dialyzer under conditions of a lower convective volume, Qi 50 mL/min (3 L/h), and specially with Qi 75 mL/min (4.5 L/h), similar efficacy and safety were obtained in comparison to the FX CorAL dialyzer in the auto-substitution HDF conditions and with good dialysate albumin losses.

There is currently sufficient scientific evidence of the superiority of post-dilution HDF versus high-flux hemodialysis in terms of the overall and cardiovascular survival [18,19,20,21]. The outcomes achieved using these new hydrophilic high-flux dialyzers validate that their application in the HD modality yields inferior results compared to HDF.

The pharmaceutical sector is consistently innovating dialyzers to enhance their efficacy and tailor them to various dialysis modalities. In recent years, there has been a growing focus on the water’s proximity to the membrane materials due to the development of materials that inhibit adsorption. The polysulfone membrane NV, developed to mitigate platelet activation, entails modifying the membrane surface’s hydrophilicity by incorporating a new hydrophilic polymer on the inner surface of the hollow fiber membrane, which is composed of polysulfone and PVP. This approach inhibits platelet adhesion, reduces the blood pressure drop, and diminishes inflammation [22]. Similarly, the helixone hydro CorAL dialyzer features a hydrophilic membrane that combines polysulfone and PVP with small quantities of tocopherol to stabilize the blood-side surface of the membrane. This combination facilitates the development of a more robust hydrophilic layer, thereby enhancing hemocompatibility, resulting in reduced protein adsorption and lower coagulation activation [12,13,14].

The behavior of this newly developed Toraylight NV dialyzer poses a dilemma in relation to its classification. In the Japanese classification [23], this filter could be classified as type IV or V super high-flux (SHF). However, in the European classification, it could not be classified as a medium cut-off (MCO) dialyzer because the performance in HD is much lower than that obtained with post-dilution HDF, in comparison with the dialyzers currently recognized in this group, such as the Theranova [24,25], Phylther SD [24], Elisio HX [25], Vie-X [25], or FDY GW [26]. On the other hand, the Toraylight NV 2.1 has not behaved like high-flux dialyzers suitable for both HD and HDF since in post-dilution HDF with auto-substitution (a situation where the maximum convective volume is reached), it has high dialysate albumin losses, so the convective volume should be limited when using it. Further dialyzers with these characteristics may be developed in the coming years, so they should be identified and classified as high-flux dialyzers with a need to limit the convection.

There are few published studies on new-generation hydrophilic high-flux dialyzers. In 2015, Hidaka et al. [11] found that, in the HD modality, the Toraylight NV improved the level of platelet-derived microparticles and endothelial function in comparison with a polysulfone membrane. In 2016, Kakuta et al. assessed the removal performance of the Toraylight NV dialyzer vs. polysulfone dialyzers, also in HD, concluding that they were comparable in removing IL-6 and β2-microglobulin but also suggesting that the NV dialyzer induced a lower release of IL-6, which would reduce the risk of erythropoiesis-stimulating agent hyporesponsiveness. The TRIATHRON 1 study [27] evaluated the antithrombogenic effect of the Toraylight NV to assess the minimum amount of anticoagulant needed to safely perform a 4-h HD treatment.

There are scarce published articles on the hydrophilic membrane in the HDF modality. A randomized clinical trial by Ehlerding et al. [28], using the FX CorAL 600 in post-dilution HDF (17.4 L), achieved a 70% RR of β2-microglobulin. The PERFORM study [29], using the FX CorAL 600 with a replacement volume of 25.4 L, obtained an RR of 85% for β2-microglobulin and 61% for myoglobin. In a previous study by Maduell et al. [15], using the FX CorAL 600 with a replacement volume of 32.8 L, RRs of 85% for β2-microglobulin and 71% for myoglobin were obtained. Kislikova et al. [30] compared the Toraylight NV 2.1 with the FX1000, both with a similar replacement volume (30 L), and obtained RRs of 79% vs. 83% for β2-microglobulin, and 76% vs. 72% for myoglobin, respectively.

The present study is the first to compare the two hydrophilic dialyzers currently available, the Toraylight NV and the FX CorAL, across a wide range of molecular weight molecules, including PBUTs and the dialysate albumin loss. In it, both treatments with the same replacement volume achieved similar β2-microglobulin, indoxyl sulfate, and p-cresyl sulfate RRs, while the myoglobin, κFLC, prolactin, α1-microglobulin, α1-acid glycoprotein, and λFLC RRs were higher with the Toraylight NV 2.1 vs. the FX CorAL 800, differences summarized in a single value with the GRS. Likewise, the different Toraylight NV treatment sessions with lower convective volumes reaffirmed the importance of the convective volume in enhancing the efficacy. In fact, the Toraylight NV 2.1 in HDF at Qi 75 mL/min results are similar to those of the FX CorAL 800 in auto-substitution HDF.

The safety of dialyzers is ensured by restricting the pore sizes to limit the albumin losses to below 5 g per session [31,32]. Although no studies with the Toraylight NV dialyzer have quantified the dialysate albumin loss, it should be underlined that the few studies involving this filter have been carried out in HD treatment and, thus, with a low risk of albumin loss. However, knowing that the in vitro albumin sieving coefficient is 0.005, greater than that of the FX CorAL (SC < 0.001), we decided to evaluate different sessions by increasing the convective volume. The best correlation observed was between the albumin loss and the Qi, and although there is great individual variability, it seems advisable not to activate the automatic self-substitution system to reach the maximum convective volume; rather, a manual prescription that must not exceed a Qi of 75 mL/min or an alternative FF that must not exceed 25%. An extended follow-up should be performed to rule out a progressive decrease in the pre-dialysis albumin.

A limitation of this study is that effectiveness was evaluated solely through the calculation of uremic toxin removal using the RRs, without long-term clinical follow-up with the dialyzers. However, the advantage of assessing effectiveness with the RRs lies in their ability to incorporate the parameters of each dialyzer into the final result. Dialysis therapy performance typically centers on solute removal, and the quality of such therapies should be evaluated through hard-outcome studies such as randomized control trials [33].

## 4. Conclusions

The new Toraylight dialyzer has great potential for efficacy but should be used at the optimal convective volume to avoid excessive albumin loss and potential clinical safety implications. Based on these results, the use of the Toraylight NV 2.1 dialyzer in post-dilution HDF at a controlled Qi not exceeding 75 mL/min or an FF lower 25% would be recommended to ensure adequate performance and compliance with safety guarantees. However, longer-term studies will be necessary to confirm these recommendations.

## Figures and Tables

**Table 1:** In vitro dialyzer performance.

	Toraylight NV-21U	FX CorAL 800
Membrane	Polysulfone (Hydrolink membrane)	Helixone hydro (polysulfone)
KUF (mL/h/mmHg)	52	73
Wall thickness	40	35
Inner diameter	200	210
SC ß_2_-microglobulin	0.93	1.00
SC myoglobin	0.55	0.60
SC albumin	0.005	<0.001
Surface (m^2^)	2.1	2.0
Sterilization	Gamma ray	Steam

KUF: ultrafiltration coefficient; SC: sieving coefficient.

**Table 2:** Comparison of the dialysis parameters in the five study sessions.

Variable	Toraylight NV HD	Toraylight NV HDF 50 mL/min	Toraylight NV HDF 75 mL/min	Toraylight NV HDF Auto-Sub	FX Coral HDF Auto-Sub
Blood processed (L)	123.3 ± 8.87	122.8 ± 8.87	123.5 ± 8.25	122.8 ± 9.17	123.2 ± 10.5
Recirculation (%)	15.8 ± 5.0	16.0 ± 4.8	16.8 ± 5.5	15.5 ± 4.9	15.5 ± 4.1
Real dialysis time (min)	285.2 ± 17.3 ^a^	284.2 ± 17.6	284.3 ± 17.6 ^a^	281.6 ± 17.6	282.9 ± 17.4
Arterial pressure (mmHg)	−228 ± 39	−221 ± 42	−225 ± 31	−216 ± 33	−218 ± 30
Venous pressure (mmHg)	208 ± 29	204 ± 28	205 ± 33	199 ± 31	201 ± 27
TMP (mmHg)	25 ± 3 ^b^	51 ± 11 ^b^	71 ± 11 ^b^	159 ± 46	177 ± 35
Initial hematocrit (%)	28.75 ± 4.84	28.67 ± 4.63	29.84 ± 4.68	28.89 ± 4.96	28.92 ± 4.73
Final hematocrit (%)	32.46 ± 5.82	32.11 ± 4.73	34.76 ± 4.84	33.58 ± 6.15	32.76 ± 5.94
Initial weight (kg)	70.04 ± 12.16	69.82 ± 12.25	70.43 ± 12.47	70.08 ± 12.19	70.25 ± 12.00
Final weight (kg)	68.21 ± 11.98	68.14 ± 12.04	68.44 ± 12.12	68.13 ± 11.99	68.31 ± 11.72
Weight gain (kg)	1.83 ± 0.89	1.68 ± 0.91	1.98 ± 0.89	1.95 ± 0.84	1.94 ± 0.74
Convective volume (L)	2.33 ± 0.89 ^b^	16.53 ± 1.48 ^b^	23.55 ± 1.82 ^b^	36.08 ± 6.50	35.89 ± 5.77
Filtration fraction (%)	NA	13.5 ± 1.4 ^b^	19.1 ± 1.1 ^b^	29.8 ± 3.9	29.1 ± 3.1

^a^ *p* < 0.05 vs. Toraylight HDF auto-sub; ^b^ *p* < 0.001 vs. all.

## Data Availability

The data that support the findings of this study are available from the corresponding author upon reasonable request.

## References

[1] Wang H., Yu T., Zhao C., Du Q. (2009). Improvement of hydrophilicity and blood compatibility on polyethersulfone membrane by adding polyvinylpyrrolidone. Fibers Polym..

[2] Jiang J., Zhu L., Zhu L., Zhang H., Zhu B., Xu Y. (2013). Antifouling and antimicrobial polymer membranes based on bioinspired polydopamine and strong hydrogen-bonded poly(N-vinyl pyrrolidone). ACS Appl. Mater. Interfaces.

[3] Zhu L., Song H., Wang J., Xue L. (2017). Polysulfone hemodiafiltration membranes with enhanced antifouling and hemocompatibility modified by poly(vinyl pyrrolidone) via in situ cross-linked polymerization. Mater. Sci. Eng. C.

[4] Sato Y., Horiuchi H., Fukasawa S., Takesawa S., Hirayama J. (2021). Influences of the priming procedure and saline circulation conditions on polyvinylpyrrolidone in vitro elution from polysulfone membrane dialyzers. Biochem. Biophys. Rep..

[5] Rodríguez-Sanz A., Sánchez-Villanueva R., Domínguez-Ortega J., Álvarez L., Fiandor A., Nozal P., Sanz P., Pizarro-Sánchez M.-S., Andrés E., Cabezas A. (2022). Characterization of hypersensitivity reactions to polysulfone hemodialysis membranes. Ann. Allergy Asthma Immunol..

[6] Sánchez-Villanueva R.J., González E., Quirce S., Díaz R., Álvarez L., Menéndez D., Rodríguez-Gayo L., Bajo M.A., Selgas R. (2014). Hypersensitivity reactions to synthetic haemodialysis membranes. Nefrologia.

[7] Alvarez-de Lara M.A., Martín-Malo A. (2014). Hypersensitivity reactions to synthetic haemodialysis membranes an emerging issue?. Nefrologia.

[8] Delgado-Córdova M., Blanco N., Azaña N. (2018). Hypotension in hemodialysis secondary to a reaction to synthetic membranes. Nefrologia.

[9] Yamaka T., Ichikawa K., Saito M., Watanabe K., Nakai A., Higuchi N., Igarashi N., Yoshimoto H. (2014). Biocompatibility of the new anticoagulant dialyzer Toraylight NV. Sci. Postprint.

[10] Hidaka S., Kobayashi S., Maesato K., Mochida Y., Ishioka K., Oka M., Moriya H., Ohtake T., Nomura S. (2015). Hydrophilic polymer-coated polysulfone membrane improves endothelial function of hemodialysis patients: A pilot study. J. Clin. Nephrol. Res..

[11] Kakuta T., Komaba H., Takagi N., Takahashi Y., Suzuki H., Hyodo T., Nagaoka M., Tanaka R., Iwao S., Ishida M. (2016). A prospective multicenter randomized controlled study on interleukin-6 removal and induction by a new hemodialyzer with improved biocompatibility in hemodialysis patients: A pilot study. Ther. Apher. Dial..

[12] Melchior P., Erlenkötter A., Zawada A.M., Delinski D., Schall C., Stauss-Grabo M., Kennedy J.P. (2021). Complement activation by dialysis membranes and its association with secondary membrane formation and surface charge. Artif. Organs.

[13] Hayama M., Yamamoto K.-I., Kohori F., Sakai K. (2004). How polysulfone dialysis membranes containing polyvinylpyrrolidone achieve excellent biocompatibility?. J. Memb. Sci..

[14] Zhu L., Song H., Zhang D., Wang G., Zeng Z., Xue Q. (2017). Negatively charged polysulfone membranes with hydrophilicity and antifouling properties based on in situ cross-linked polymerization. J. Colloid. Interface Sci..

[15] Maduell F., Broseta J.J., Rodríguez-Espinosa D., Rodas L.M., Gómez M., Arias-Guillén M., Fontseré N., Vera M., Salgado M.d.C., Rico N. (2024). Comparison of efficacy and safety of the new generation helixone dialyzers. Nefrologia.

[16] Bergström J., Wehle B. (1987). No change in corrected β2-microglobulin concentration after cuprophane hemodialysis. Lancet.

[17] Maduell F., Rodas L., Broseta J.J., Gomez M., Xipell M., Guillen E., Montagud-Marrahi E., Arias-Guillén M., Fontseré N., Vera M. (2019). Medium Cut-Off Dialyzer versus Eight Hemodiafiltration Dialyzers: Comparison Using a Global Removal Score. Blood Purif..

[18] Maduell F., Moreso F., Pons M., Ramos R., Mora-Macià J., Carreras J., Soler J., Torres F., Campistol J.M., Martinez-Castelao A. (2013). High-efficiency postdilution online hemodiafiltration reduces all-cause mortality in hemodialysis patients. J. Am. Soc. Nephrol..

[19] Mercadal L., Franck J.E., Metzger M., Ureña Torres P., de Cornelissen F., Edet S., Béchade C., Vigneau C., Drüeke T., Jacquelinet C. (2015). Hemodiafiltration versus hemodialysis and survival in patients with ESRD: The French renal epidemiology and information network (REIN) registry. Am. J. Kidney Dis..

[20] See E.J., Hedley J., Agar J.W.M., Hawley C.M., Johnson D.W., Kelly P.J., Lee V.W., Mac K., Polkinghorne K.R., Rabindranath K.S. (2019). Patient survival on haemodiafiltration and haemodialysis: A cohort study using the Australia and New Zealand Dialysis and Transplant Registry. Nephrol. Dial. Transplant..

[21] Blankestijn P.J., Vernooij R.W.M., Hockham C., Strippoli G.F.M., Canaud B., Hegbrant J., Barth C., Covic A., Cromm K., Cucui A. (2023). CONVINCE Scientific Committee Investigators. Effect of Hemodiafiltration or Hemodialysis on Mortality in Kidney Failure. N. Engl. J. Med..

[22] Oshihara W., Ueno Y., Fujieda H., Kawanishi H., Takemoto Y. (2017). New Polysulfone Membrane Dialyzer, NV, with Low-Fouling Antithrombotic Properties. Scientific Aspects of Dialysis Therapy: JSDT/ISBP Anniversary Edition.

[23] Abe M., Masakane I., Wada A., Nakai S., Nitta K., Nakamoto H. (2021). Dialyzer classification and mortality in hemodialysis patients: A 3-year nationwide cohort study. Front. Med..

[24] Belmouaz M., Bauwens M., Hauet T., Bossard V., Jamet P., Joly F., Chikhi E., Joffrion S., Gand E., Bridoux F. (2020). Comparison of the removal of uraemic toxins with medium cut-off and high-flux dialysers: A randomized clinical trial. Nephrol. Dial. Transplant..

[25] Maduell F., Broseta J.J., Rodríguez-Espinosa D., Del Risco J., Rodas L.M., Arias-Guillén M., Vera M., Fontseré N., Salgado M.d.C., Rico N. (2022). Comparison of four medium cut-off dialyzers. Clin. Kidney J..

[26] García-Prieto A., de la Flor J.C., Coll E., Iglesias E., Reque J., Valga F. (2023). Expanded hemodialysis: What’s up, Doc?. Clin. Kidney J..

[27] Ronco C., Brendolan A., Nalesso F., Zanella M., Cal M.D., Corradi V., Virzì G.M., Ferrari F., Garzotto F., Lorenzin A. (2017). Prospective, randomized, multicenter, controlled trial (TRIATHRON 1) on a new antithrombogenic hydrophilic dialysis membrane. Int. J. Artif. Organs.

[28] Ehlerding G., Erlenkötter A., Gauly A., Griesshaber E., Kennedy J., Rauber L., Ries W., Schmidt-Gürtler H., Stauss-Grabo M., Wagner S. (2021). Performance and Hemocompatibility of a Novel Polysulfone Dialyzer: A Randomized Controlled Trial. Kidney360.

[29] Ehlerding G., Ries W., Kempkes-Koch M., Ziegler E., Erlenkötter Zawada A.M., Kennedy J.P., Ottillinger B., Stauss-Grabo M., Lang T. (2022). Randomized comparison of three high-flux dialyzers during high-volume online hemodiafiltration-the comPERFORM study. Clin. Kidney J..

[30] Kislikova M., Vega A., Verde E., Abad S., Vaca M., Acosta A., González A., Bascuñana A., Mijailova A., Nava C. (2024). Depurative capacity toward medium molecules of the dialyzer Toray NV-U^®^ HydrolinkTM: A new hydrophilic membrane to perform online hemodiafiltration. Int. J. Artif. Organs.

[31] Potier J., Queffeulou G., Bouet J. (2016). Are all dialyzers compatible with the convective volumes suggested for postdilution online hemodiafiltration?. Int. J. Artif. Organs.

[32] Boschetti-De-Fierro A., Voigt M., Storr M., Krause B. (2015). MCO Membranes: Enhanced Selectivity in High-Flux Class. Sci. Rep..

[33] Masakane I., Sakurai K. (2018). Current approaches to middle molecule removal: Room for innovation. Nephrol. Dial. Transplant..

